# Transcriptomic and metabolomic analyses of the ovaries of Taihe black-bone silky fowls at the peak egg-laying and nesting period

**DOI:** 10.3389/fgene.2023.1222087

**Published:** 2023-10-09

**Authors:** Xin Xiang, Xuan Huang, Jianfeng Wang, Haiyang Zhang, Wei Zhou, Chunhui Xu, Yunyan Huang, Yuting Tan, Zhaozheng Yin

**Affiliations:** ^1^ Animal Science College, Zhejiang University, Hangzhou, China; ^2^ Hangzhou Original Seed Farm, Hangzhou, China

**Keywords:** Taihe black-bone silky fowl, ovary, transcriptome, metabolome, reproductive performance

## Abstract

The poor reproductive performance of most local Chinese chickens limits the economic benefits and output of related enterprises. As an excellent local breed in China, Taihe black-bone silky fowl is in urgent need of our development and utilization. In this study, we performed transcriptomic and metabolomic analyses of the ovaries of Taihe black-bone silky fowls at the peak egg-laying period (PP) and nesting period (NP) to reveal the molecular mechanisms affecting reproductive performance. In the transcriptome, we identified five key differentially expressed genes (DEGs) that may affect the reproductive performance of Taihe black-bone silky fowl: BCHE, CCL5, SMOC1, CYTL1, and SCIN, as well as three important pathways: the extracellular region, Neuroactive ligand-receptor interaction and Cytokine-cytokine receptor interaction. In the metabolome, we predicted three important ovarian significantly differential metabolites (SDMs): LPC 20:4, Bisphenol A, and Cortisol. By integration analysis of transcriptome and metabolome, we identified three important metabolite-gene pairs: “LPC 20:4-BCHE”, “Bisphenol A-SMOC1”, and “Cortisol- SCIN”. In summary, this study contributes to a deeper understanding of the regulatory mechanism of egg production in Taihe black-bone silky fowl and provides a scientific basis for improving the reproductive performance of Chinese local chickens.

## 1 Introduction

Eggs are an important food resource that contains a large amount of essential nutrients for the human body. Egg production is an important indicator of the reproductive performance of chickens, which affects the profits and productivity of the laying hen industry (Mu et al., 2021). The ovary is a key organ of the reproductive system of poultry and is critical to their reproductive performance. In recent years, most studies have focused on the ovaries of mammals, and relatively few studies have been conducted on the ovaries of poultry (Lin et al., 2021). Therefore, in-depth studies on poultry ovaries further provide a theoretical basis for the egg-laying mechanism of poultry.

Improving the reproductive performance of poultry is an important breeding goal, but traditional breeding methods have progressed slowly and it has been difficult to identify specific genetic improvements (Biscarini et al., 2010). We performed transcriptome sequencing and metabolome sequencing of ovaries from Taihe black-bone silky fowls, and performed integration analysis of the transcriptome and metabolome to reveal the molecular mechanisms involved in egg production performance. Transcriptome sequencing technology is a high-throughput sequencing technology that allows differential gene analysis at the genome-wide level; metabolomics is closely related to phenomics and can reflect the physiological state of an organism more directly and accurately (Li et al., 2022). Lin et al. (2021) performed transcriptome sequencing of Muscovy duck ovaries and predicted six genes that may regulate ovulation: CTNNB1, IGF1, FOXO3, HSPA2, PTEN, and SMC4; and four important pathways: the Adhesion-related pathway, mTOR pathway, TGF-β signaling pathway and FoxO signaling pathway. Yuan et al., 2020) performed a metabolomic analysis of stearoyl-CoA desaturase (SCD) during goose follicle development and identified cholesterol and pantothenic acid as potential biomarker metabolites of goose granulosa cells. Transcriptomic and metabolomic integration analysis can correlate genes and metabolites (Tohge et al., 2005). Therefore, the use of transcriptomic and metabolomic integration analysis can provide a more comprehensive understanding of ovarian performance in the Taihe black-bone silky fowl. Wu et al. (2022) performed a transcriptomic and metabolomic integration analysis to reveal the effect of light supplementation on sternal calcification in ducks. (Ma et al. (2022) performed a transcriptomic and metabolomic integration analysis to reveal the modulation of fructo-oligosaccharide on ileum metabolism of Taiping chickens.

Nesting is an instinct of hens to reproduce, and during nesting, the ovarian function of hens will degenerate, and nesting is common in Chinese local chickens. The Taihe black-bone silky fowl is a Chinese local breed originated from Wangbantu village, Taihe County, Jiangxi Province, with good meat quality and flavor, which is worthy of our in-depth study (Mi et al., 2018). Most Chinese local breeds of chickens have low egg production, and their reproductive performance needs to be improved. In this study, we performed transcriptomic and metabolomic integration analyses on ovaries of Taihe black-bone silky fowls at the peak egg-laying period and nesting period, and identified key differentially expressed genes, significantly differential metabolites and related pathways that may affect the reproductive performance of Taihe black-bone silky fowl, and we also predicted important metabolite-gene pairs. These findings will provide a new perspective on the molecular mechanism of ovarian egg production in the Taihe black-bone silky fowl, as well as a theoretical basis for improving its reproductive performance.

## 2 Materials and methods

### 2.1 Animal and sample collection

Twelve Taihe black-bone silky fowls were purchased from the Taihe county in the Jiangxi province from the Taihe Aoxin black-bone silky fowl Development Co. Among them, six each were peak egg-laying period (203-day-old chickens, PP) and nesting period (394-day-old chickens, NP), and all sample chickens were randomly selected. Ovarian tissues from these 12 chickens were collected, rinsed with PBS (phosphate buffer saline), and immediately preserved in liquid nitrogen.

### 2.2 Ethical statement

All the animals used in this experiment conform to the standards in the Chinese Animal Welfare Guidelines and are approved by the Animal Experimentation Ethics Committee of Zhejiang University (approval number:ZJU20190149).

### 2.3 Transcriptome sequencing and data analysis

Beijing Novozymes Technology Co., Ltd. was responsible for the transcriptome sequencing and library construction of the collected Taihe black-bone silky fowl ovaries. Subsequently, the illumina NovaSeq 6000 sequencing platform was used to sequence and construct the gene library. The raw data were processed to obtain clean data to ensure the quality and reliability of data analysis. For the clean data, Q20, Q30 and GC content were calculated, and we used HISAT2 v2.0.5 to construct the index of the reference genome, while comparing the clean reads with the reference genome. FeatureCounts (1.5.0-p3) is used to calculate the number of reads mapped to each gene and FPKM. Differential expression analysis was performed using DESeq2 software (1.20.0), and those with *p* < 0.05 were identified as differentially expressed genes by statistical procedures; *p*-values were adjusted using Benjamini and Hochberg methods to control the incidence of errors. GO (Gene Ontology) enrichment analysis and KEGG (Kyoto Encyclopedia of Genes and Genomes) enrichment analysis of differentially expressed genes were performed by clusterProfiler (3.8.1) software. GO is a comprehensive database describing gene functions, and KEGG is a comprehensive database integrating genomic, chemical and systematic functional information.

### 2.4 Metabolome sequencing and data analysis

Beijing Novozymes Technology Co., Ltd. was responsible for the metabolomic analysis of the collected ovaries of Taihe black-bone silky fowls. We used Vanquish UHPLC chromatograph and Q Exactive™ HF mass spectrometer for LC-MS/MS analytical processing. Compound Discoverer 3.1 (CD3.1; Thermo Fisher) was used for data pre-processing and metabolite identification. The identified metabolites were annotated using the KEGG database, HMDB database and LIPIDMaps database. Partial least squares discriminant analysis (PLS-DA) and principal component analysis (PCA) were performed on the processed data using metaX software to calculate VIP values; and based on t-tests to calculate *p*-values and fold change (FC value). In order to identify the significantly different metabolite (SDM) between the PP and NP, the Variable Importance in the Projection (VIP) of the first principal component of the PLS-DA model, the difference fold change (FC) of each metabolite in the comparison group, and the *p*-value obtained by *t*-test were used to identify the significantly different metabolite. The screening criteria for significantly differential metabolites were VIP >1, *p*-value <0.05 and FC ≥ 2 or FC ≤ 0.5. Cluster heat maps of significantly differential metabolites were drawn using R language and correlation analysis was performed. Enrichment analysis of metabolites was performed using the KEGG database.

### 2.5 Transcriptome and metabolome integration analysis

Based on Pearson correlation coefficient, correlation analysis was performed on differentially expressed genes and significantly differential metabolites to measure the degree of association between them. When the correlation coefficient is less than 0, it is called negative correlation; when it is greater than 0, it is called positive correlation. We mapped all the differentially expressed genes and significantly differential metabolites obtained simultaneously to the KEGG pathway database to determine their common pathway information.

## 3 Results

### 3.1 Transcriptomic analysis of differentially expressed genes

**FIGURE 1:**
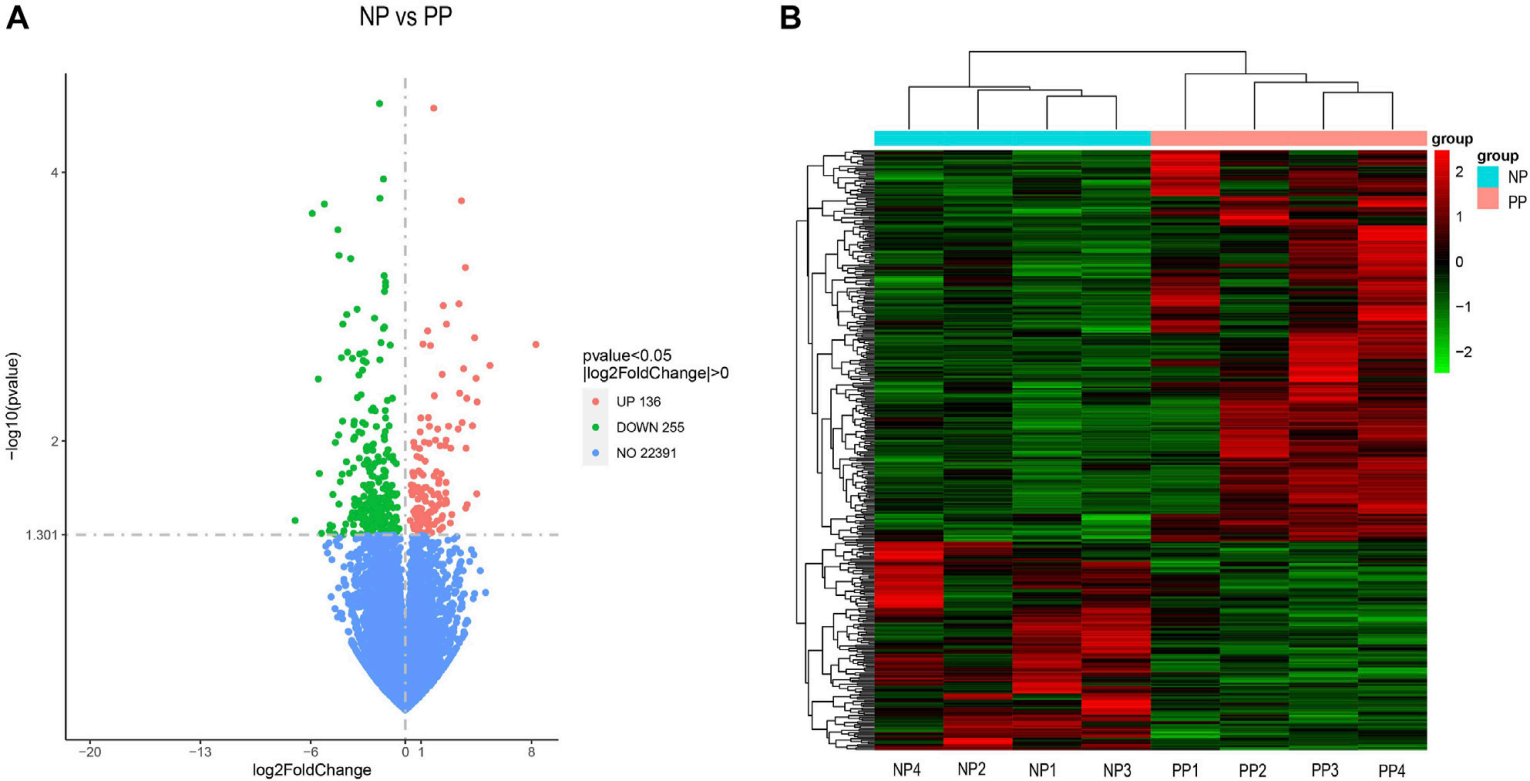
Volcano plot of differentially expressed genes **(A)**, horizontal coordinate *X*-axis indicates the log2FoldChange, vertical coordinate *Y*-axis indicates the significance level of the difference (-log10 *p*-value). Red dots: upregulated genes; green dots: downregulated genes; blue dots: non-differential genes. Hierarchical clustering analysis of the DEGs **(B)**.

A total of 391 differentially expressed genes (DEGs) were identified by transcriptome analysis of the ovaries of Taihe black-bone silky fowls at the peak egg-laying period (PP) and nesting period (NP). The threshold for screening was *p* < 0.05. Among them, 136 genes were upregulated and 255 genes were downregulated. The following are the volcano plot of differentially expressed genes and hierarchical cluster analysis (Figures 1A, 1B; Supplementary Table S1). By relative expression levels of differentially expressed genes and pathways related to reproductive performance, we screened five differential expressed genes that may affect the egg-laying performance of Taihe black-bone silky fowl, they are BCHE,CCL5,SMOC1,CYTL1, and SCIN.

### 3.2 Transcriptome GO and KEGG enrichment pathway analysis

**FIGURE 2:**
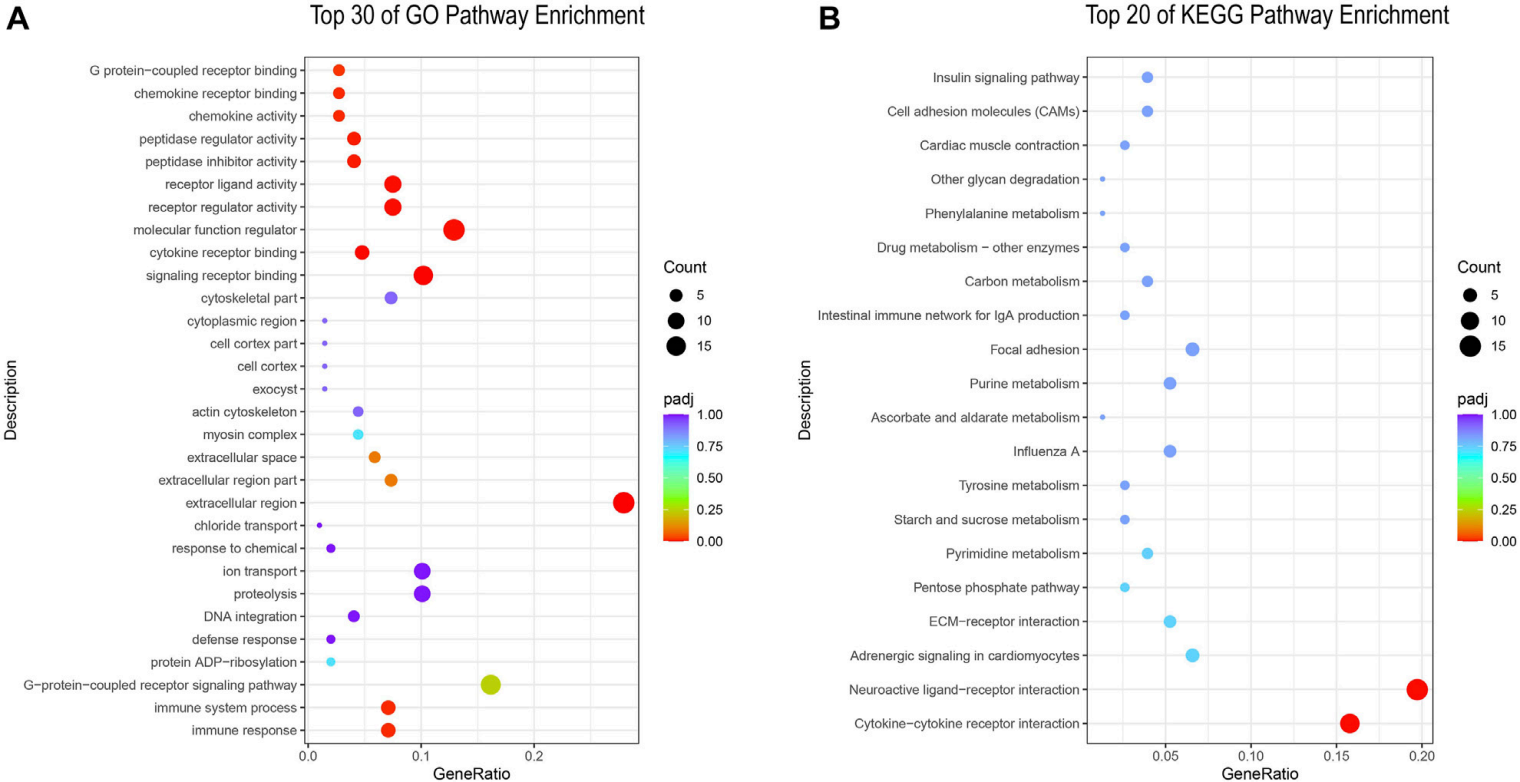
**(A)** GO enrichment analysis of DEGs; **(B)** KEGG enrichment analysis of DEGs.

In order to gain a deeper understanding of ovarian development, we performed GO and KEGG (pathway enrichment analysis on DEGs in the PP and NP. In the GO pathway enrichment, a total of 314 differentially expressed genes were enriched into 330 pathways, and we listed the top 30 GO-enriched pathways (Figure 2A; Supplementary Table S2). Among them, molecular function regulator, signaling receptor binding and extracellular region are the three most enriched pathways, and extracellular region is the most representative pathway. In KEGG pathway enrichment, a total of 76 differentially expressed genes were enriched into 72 pathways, and we listed the top 20 KEGG-enriched pathways (Figure 2B; Supplementary Table S3). Among them, Neuroactive ligand-receptor interaction and Cytokine-cytokine receptor interaction were the two most enriched and representative pathways.

### 3.3 Quality control and partial least squares discriminant analysis (PLS-DA) in metabolomics

**FIGURE 3:**
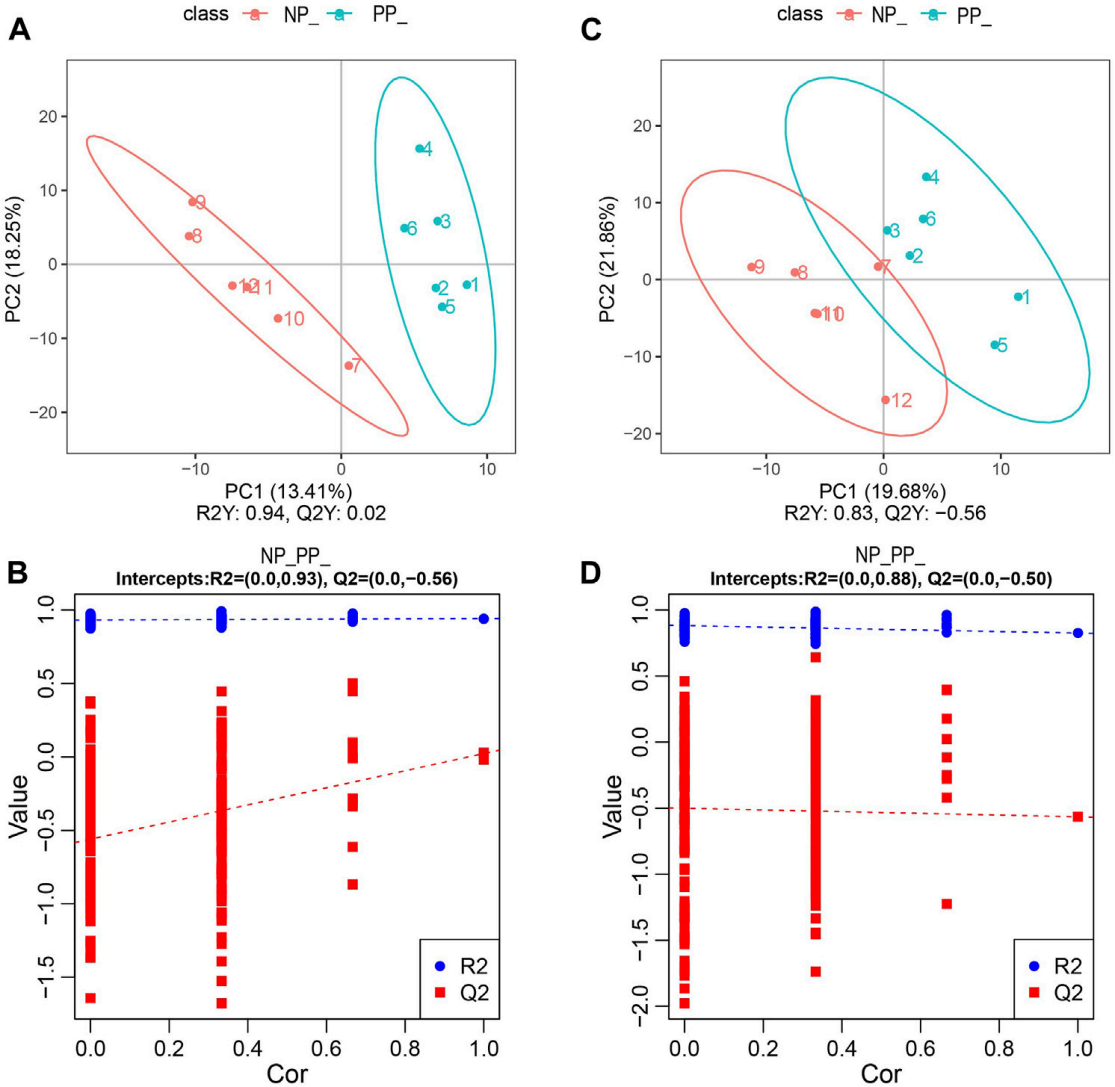
**(A)** PLS-DA analysis in the positive model; **(B)** PLS-DA alignment test in the positive model; **(C)** PLS-DA analysis in the negative model; **(D)** PLS-DA alignment test in the negative model.

In this study, we performed metabolomic analyses of ovaries from the PP and NP of the Taihe black-bone silky fowl. By partial least squares discriminant analysis (PLS-DA), there was a significant difference between the PP and NP(Figures 3A, C). At the same time, the parameters R2 and Q2 of the PLS-DA model were replaced with 200 trials, and their regression lines could be obtained based on the R2 and Q2 values after 200 disruptions and modeling, and the PLS-DA model was not overfitted when the R2 value was greater than the Q2 value and the intercept of the Q2 regression line with the *Y*-axis was less than 0, indicating that our data were reliable (Figures 3B, D).

### 3.4 Metabolomics differential metabolite analysis

**FIGURE 4:**
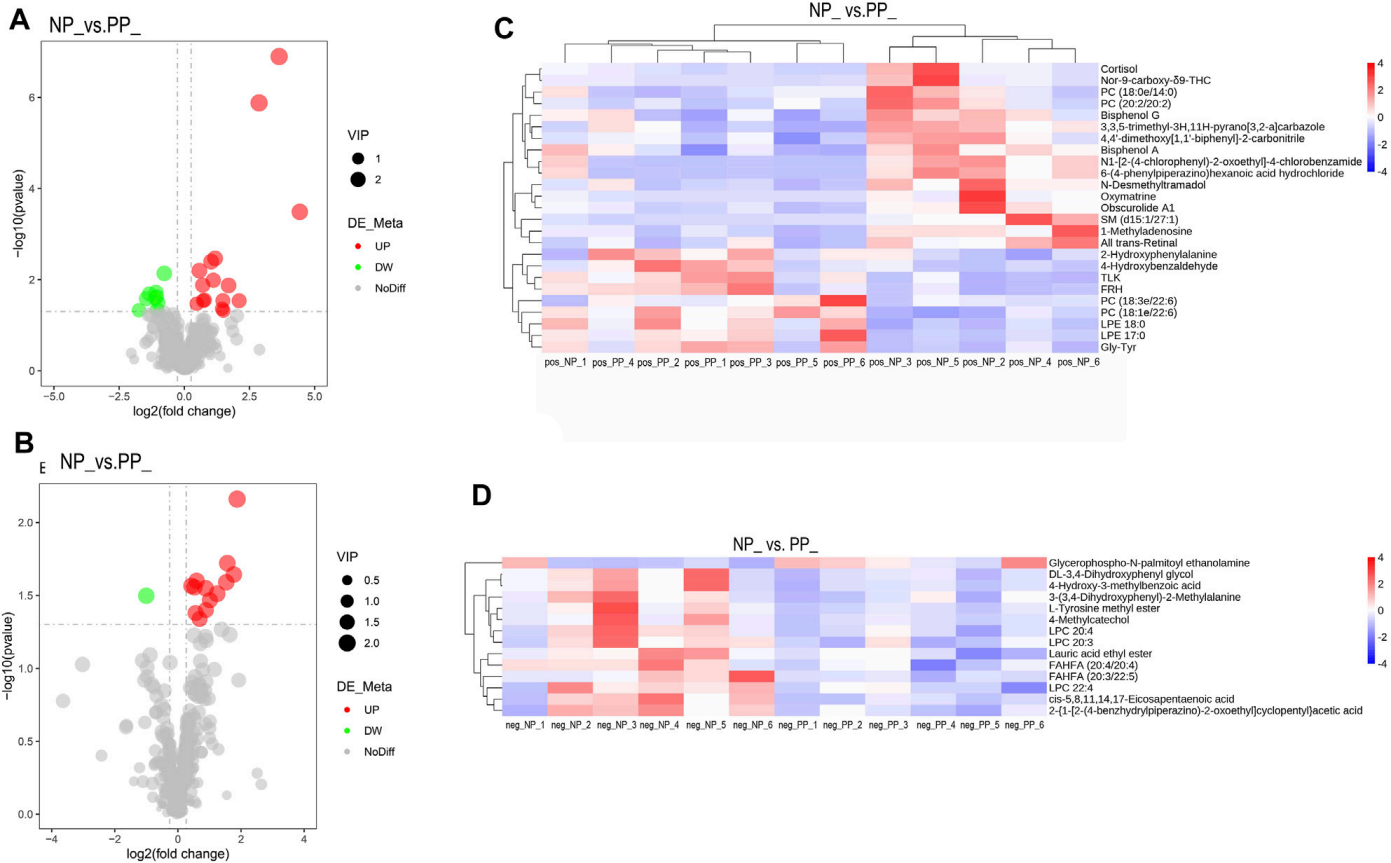
Volcano plots of differential metabolites in PP and NP, horizontal coordinates indicate log2FoldChange, vertical coordinates indicate -log10*p*-value, red dots indicate significantly upregulated metabolites, green dots indicate significantly downregulated metabolites, **(A)** positive model of differential metabolites, **(B)** negative model of differential metabolites. **(C)** Heat map of significantly different metabolite clusters in the positive model, and **(D)** heat map of significantly different metabolite clusters in the negative model, with vertical clusters representing different metabolites and horizontal clusters representing different samples.

By setting the thresholds VIP >1.0, FC > 1.2 or FC < 0.833 and *p* < 0.05, a total of 39 SDMs were identified, of which 25 SMDs in the positive model and 14 SDMs in the negative model. We screened three significantly differential metabolites that may affect the egg production performance of Taihe black-bone silky fowl, they are LPC 20:4, Bisphenol A, and Cortisol. The following are the volcano map and hierarchical cluster analysis of SDMs (Figures 4A–D; Supplementary Tables S4, S5).

### 3.5 Integrative analysis of transcriptomics and metabolomics

**FIGURE 5:**
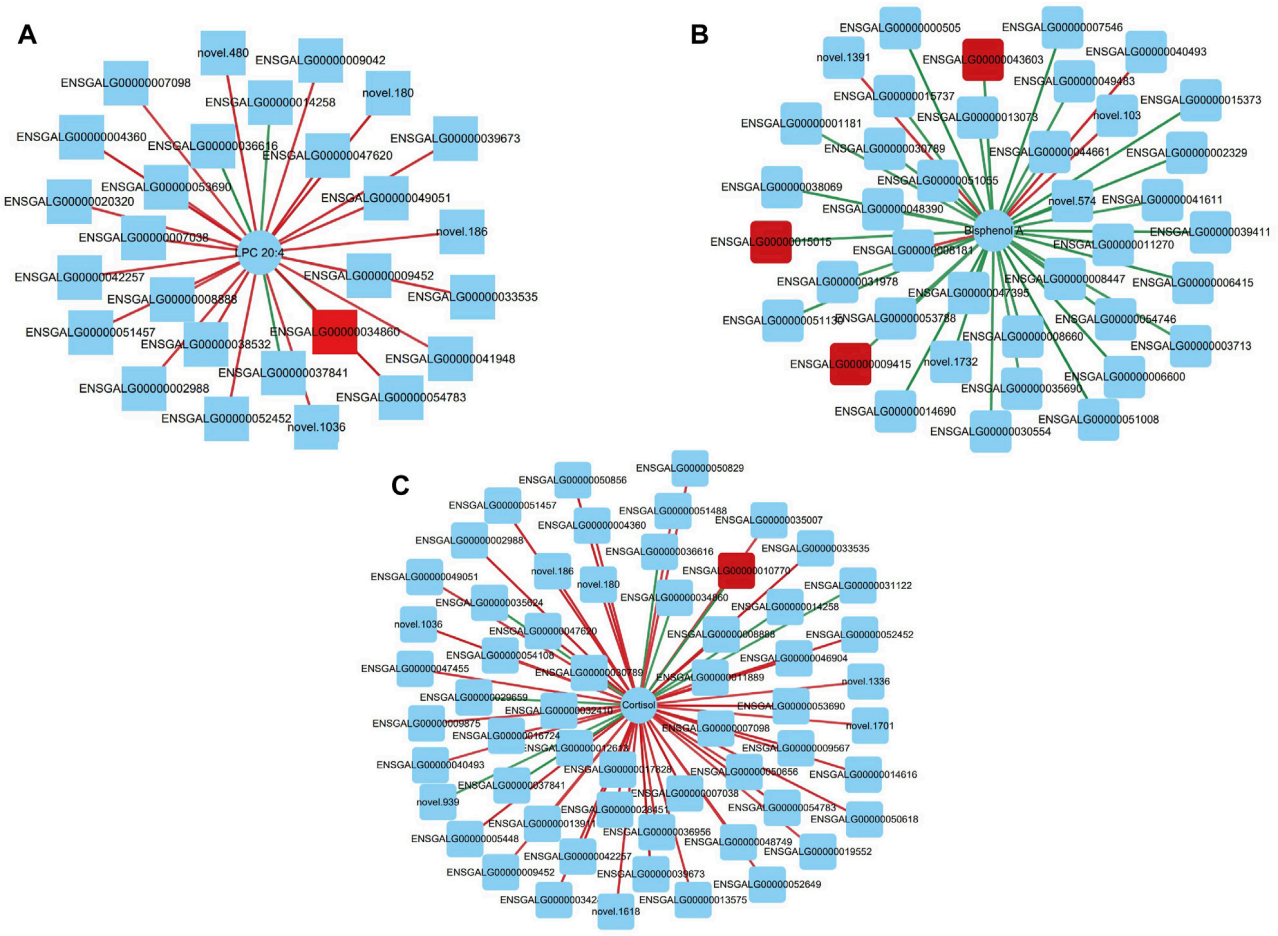
**(A)** Correlation network diagram of LPC 20:4 and differentially expressed genes; **(B)** Correlation network diagram of Bisphenol A and differentially expressed genes; **(C)** Correlation network diagram of Cortisol and differentially expressed genes. Circles indicate significantly different metabolites, squares indicate differentially expressed genes, red lines indicate positive correlations (red squares indicate the differentially expressed genes we screened), green lines indicate negative correlations, and the thickness of the lines indicates the strength of the correlation.

Based on Pearson correlation analysis, the correlation between transcriptomic DEGs and metabolomic SDMs was revealed. When the correlation coefficient is less than 0, it is called negative correlation; when it is greater than 0, it is called positive correlation. We plotted the correlation heat map of all significantly differential metabolites and Top 100 differentially expressed genes (Supplementary Figures S1, S2). The results indicate that the transcriptome and metabolome are strongly correlated. Furthermore, we correlated specific metabolites and genes that may regulate ovarian development and reproductive performance in laying hens, searching for important metabolite-gene pairs to explore further potential roles. We considered metabolite-gene pairs that satisfied both correlation >0.8 and *p* < 0.05 as strongly correlated metabolite-gene pairs, and plotted the correlation network using Cytoscape_v3.9.1 (Figures 5A–C; Supplementary Tables S6, S7). We identified three metabolite-gene pairs that may affect egg-laying performance in Taihe black-bone silky fowl: “LPC 20:4- BCHE”, “Bisphenol A- SMOC1” and “Cortisol- SCIN”.

**FIGURE 6:**
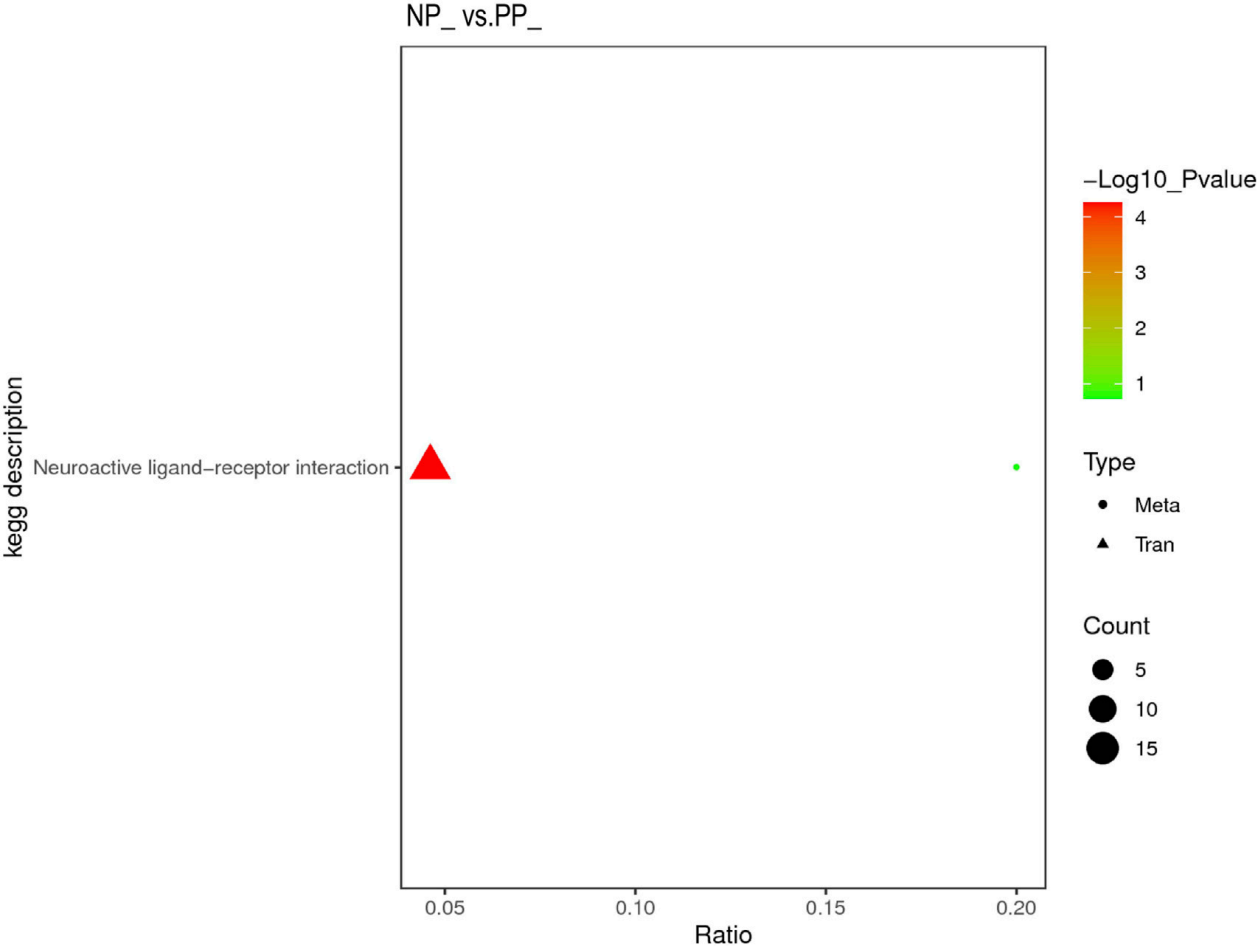
Transcriptome and metabolome integration analysis of KEGG pathway enrichment.

Both DEGs in the transcriptome and SDMs in the metabolome were significantly enriched to the Neuroactive ligand-receptor interaction pathway, indicating that Neuroactive ligand-receptor interaction is a very important pathway affecting the egg production performance of Taihe black-bone silky fowl (Figure 6).

## 4 Discussion

Eggs are an important food resource for humans, and improving egg production is an important goal for the poultry industry. The ovary is an important reproductive organ of poultry, and the health and normal development of the ovary is crucial to the egg production performance of poultry. In-depth studies on ovaries can help to further improve the egg production performance of poultry. In this study, transcriptomic and metabolomic techniques were used to study and analyze the ovaries of Taihe black-bone silky fowls at the PP and NP. We identified five differentially expressed genes, three important pathways and three significant differential metabolites that may affect the egg production performance of Taihe black-bone silky fowl. The differentially expressed genes are BCHE, CCL5, SMOC1, CYTL1, and SCIN; the important pathways are extracellular region, Neuroactive ligand-receptor interaction and Cytokine-cytokine receptor interaction; the significant differential metabolites are LPC 20:4, Bisphenol A and Cortisol. In addition, we identified three metabolite-gene pairs that may affect egg-laying performance in Taihe black-bone silky fowl, namely, “LPC 20:4-BCHE”, “Bisphenol A-SMOC1” and “Cortisol-SCIN”. We believe that our study will provide new insights into the egg-laying mechanism in poultry.

### 4.1 Transcriptomic analysis

In this study, in order to find out the key genes that affect the egg production performance of Taihe black-bone silky fowls, 391 DEGs were identified in the ovaries of Taihe black-bone silky fowls during the PP and NP. We screened five differentially expressed genes that may affect the egg production performance of Taihe black-bone silky fowls, they are *BCHE*, *CCL5*, *SMOC1*, *CYTL1*, and *SCIN*. Butyrylcholinesterase (BCHE) has several physiological functions and is an enzyme that can be involved in the cholinergic system (Glombowsky et al., 2017). The concentration of *BCHE* increases significantly in sows during parturition, suggesting that *BCHE* may help sows to complete parturition (Contreras et al., 2021). It has been shown that *BCHE* can influence embryonic developmental processes (Paraoanu et al., 2006). In addition, *BCHE* plays an important role in the development of the nervous system in poultry (Layer et al., 1991). In this study, we found that the expression of this gene was significantly higher in the PP than in the NP, it may affect egg formation through the nervous system and we predict that this gene has an important role in the egg production performance of poultry. The C-C chemokine ligand 5 (CCL5) is a member of the chemokine family, and *CCL5* has a chemotactic effect on immune cells and induces activation of immune cells to fight infection (Choi et al., 2020). It has been suggested that *CCL5* may mediate autocrine and paracrine secretion to regulate ovarian activity during ovulation (Skinner et al., 2008). In addition, *CCL5* plays an important role in the luteolysis process (Witek et al., 2020). The expression of this gene is significantly higher in the PP than in the NP and may regulate the immune response during egg production to ensure ovarian health. Secreted modular calcium-binding protein 1 (SMOC1) is an extracellular glycoprotein that is involved in a variety of physiological functions. It has been suggested that *SMOC1* may mediate cell type-specific differentiation and intercellular signaling during fetal gonadal and reproductive tract differentiation (Pazin et al., 2009). Bao et al. (2021) found that *SMOC1* has an important regulatory role in the egg production performance of muscovy duck. In addition, *SMOC1* has an important regulatory role in embryonic development (Gao et al., 2019). *SMOC1* is expressed in the zona pellucida of oocytes (Vannahme et al., 2002), and the expression of this gene is significantly higher in the PP than in the NP. We hypothesize that *SMOC1* can mediate the maturation of oocytes and has an important role in the egg production performance of poultry. Cytokine-like protein 1 (CYTL1) is a functional secreted protein. In the ovary, elevated concentrations of progesterone or estradiol lead to enhanced *CYTL1* expression; in the uterus, *CYTL1* expression is significantly enhanced in endometrial cells with increasing concentrations of progesterone and estrogen, suggesting that *CYTL1* is a candidate marker of endometrial tolerance and that upregulation of *CYTL1* leads to significant proliferation of endometrial cells (Ai et al., 2016). In addition, it has been reported that *CYTL1* can mediate the regulation of different stages of folliculogenesis (Moura et al., 2021). The tolerance of the endometrium is important for the reproduction of offspring in females, in the study, we hypothesized that the high expression of this gene during the PP contributes to ovarian maintenance and has an important role in egg production. Scinderin (SCIN)is a Ca^2+^-dependent protein belonging to the gelsolin superfamily. Sperm capacitation and acrosome reaction are key steps in mammalian fertilization, and *SCIN* is one of the key binding proteins that control this polymerization (Breitbart et al., 2005). It has been suggested that *SCIN* may have a regulatory role in the fertility of pigs (Liang et al., 2020). *SCIN* can produce circSCIN, which can bind to MiR-133 and MiR-148b, MiR-133 can regulate oocyte meiosis and MiR-148b can mediate estrogen secretion (Yao et al., 2010; Song et al., 2014; Wu et al., 2018). We speculate that *SCIN* has an important regulatory role in oocyte development and estrogen secretion. The expression of this gene was significantly higher in the PP than in the NP and may have an important role in egg production performance. In summary, the expression of these five genes was significantly higher in the PP than in theNP and may have an important role in ovarian and egg production performance. Other differentially expressed genes may also have important effects on egg production performance, and their functions will be further explored in subsequent studies.

In order to further understand the possible functions involved in DEGs, we performed GO annotation (Gene Ontology) and KEGG analysis (Kyoto Encyclopedia of Genes and Genomes) on DEGs. We screened three pathways that may affect egg production performance in Taihe black-bone silky fowls: extracellular region, Neuroactive ligand-receptor interaction and Cytokine-cytokine receptor interaction. It has been suggested that the extracellular region may have an effect on pig pregnancy (Samborski et al., 2013). Ge et al. (2017) ound that the extracellular region can mediate the maturation process of zebrafish oocytes. Sun et al. (2022) found that the extracellular region plays a key role in follicle development in chickens. In the study, the extracellular region was the most enriched and representative pathway of the GO pathway, and we hypothesized that it might have an important role in the egg production performance of the Taihe black-bone silky fowl. We found significant differences in the expression of DEGs in the Neuroactive ligand-receptor interaction between the PP and NP, with the Neuroactive ligand-receptor interaction being the most enriched pathway in the KEGG pathway. Transcriptomic studies in zebrafish (Chen et al., 2019), goats (Su et al., 2018) and pigs (Xu et al., 2015) have shown that Neuroactive ligand-receptor interactions have important effects on reproductive performance. Mu et al. (2021) found that Neuroactive ligand-receptor interactions may be the most important pathway leading to significant differences in egg production rates between high-laying and low-laying hens. In addition, it has been shown that Neuroactive ligand-receptor interactions have important effects on egg production performance in ducks (Tao et al., 2017) and geese (Ouyang et al., 2020). In the study, the Cytokine-cytokine receptor interaction pathway was significantly enriched, coinciding with a related report in the Nandan-Yao domestic chicken (Sun et al., 2021). Quan et al. (2019) found that this pathway has important effects on follicle development and pregnancy establishment in goats. In addition, transcriptome studies in pigs (Yang et al., 2018) and geese (Zhao et al., 2022) showed that Cytokine-cytokine receptor interactions have an important role in ovarian development and ovulation. Briefly, the three pathways of extracellular region, Neuroactive ligand-receptor interaction and Cytokine-cytokine receptor interaction are considered to be closely related to the reproductive performance of Taihe black-bone silky fowl and have important effects on the egg production performance of Taihe black-bone silky fowl. Some pathways that are not significantly enriched may also have important effects on egg production performance, and their functions will be further explored in subsequent studies.

### 4.2 Metabolomics analysis

Metabolomics is closer to phenomics, which is an extension of transcriptomics and proteomics, and can reflect the physiological state of an organism more directly and accurately. In the study, we identified 39 significantly different metabolites in the ovaries of Taihe black-bone silky fowl during the PP and NP, including 25 significantly different metabolites in the positive model and 14 significantly different metabolites in the negative model. We screened three significantly different metabolites that might affect the egg production performance of Taihe black-bone silky fowl: LPC 20:4, Bisphenol A, and Cortisol. LPC 20:4 is an isoform of lysophosphatidylcholine (LPC), and it has been shown that LPC not only affects the acrosome response of sperm and eggs, but also mediates paracrine actions in oocytes (Gomez-Torres et al., 2015). Yang et al. found that LPC can mediate follicular development and is a predictor of follicular development (Yang et al., 2022a). Lysophosphatidylcholine (LPC) can be converted to lysophosphatidic acid (LPA) by the action of enzymes, and LPA has important effects on the maintenance of ovarian function, embryonic development and pregnancy maintenance, which is sufficient to show the important role of LPC on female reproductive performance (Ye et al., 2008). In addition, it has been shown that LPC has an inhibitory effect on the cell viability of mouse ovarian granulosa cells (Yang et al., 2022b). From this, we inferred that LPC 20:4 may affect ovarian function in Taihe black-bone silky fowl. Bisphenol A is a chemical with endocrine disrupting properties that affects ovarian estrogen and steroid hormone secretion (Bloom et al., 2016). Bisphenol A can bind to estrogen receptor and has estrogen effect, which has certain influence on oocyte maturation (Rochester et al., 2013). Bisphenol A can affect primordial follicle formation by promoting the progression of meiosis in oocytes (Yu et al., 2018). It has been shown that Bisphenol A may adversely affect follicle formation and affect the healthy development of reproductive organs in chickens (Mentor et al., 2020; Eldefrawy et al., 2021). In addition, it has been shown that Bisphenol A may impair the reproductive adaptations of zebrafish ovaries (Biswas et al., 2020). Cortisol is a glucocorticoid with several physiological functions, such as: response to stress, regulation of apoptosis and lipid metabolism. It has been shown that Cortisol can affect folliculogenesis and oocyte maturation in cows, support embryo implantation, and improve pregnancy rates in cows (da et al., 2015; Duong et al., 2012). Lack of cortisol causes infertility in female mice, and high cortisol levels affect granulosa cell function, leading to a decrease in estradiol (Mullins et al., 2009; Prasad et al., 2016). Xiao et al. found that cortisol can protect oogenesis by promoting follicular cell survival (Xiao et al., 2022). In addition, it has been shown that cortisol can affect sexual development and reproductive function in zebrafish (Zhang et al., 2020). In conclusion, the above three significantly different metabolites may be essential metabolites in the egg-laying process of Taihe black-bone silky fowl, they may affect the health of the ovaries, the viability of ovarian granulosa cells and the process of oogenesis in Taihe black-bone silky fowl Some metabolites were not significant differential metabolites, but they may also have important effects on egg production performance, and we will explore these metabolites further in subsequent studies.

### 4.3 Transcriptome and metabolome integration analysis

We performed integrated transcriptomic and metabolomic analyses of ovaries from the PP and the NP in Taihe black-bone silky fowls. Based on Pearson correlation analysis, specific metabolites and genes that may regulate ovarian development and reproductive performance of laying hens were correlated, and important metabolite-gene pairs were searched for to explore further potential roles. LPC 20:4, Bisphenol A and Cortisol may be significant differential metabolites with important effects on egg production performance, BCHE, SMOC1 and SCIN may be differentially expressed genes with important effects on egg production performance, LPC 20:4 and BCHE, Bisphenol A and SMOC1, Cortisol and SCIN all have strongly correlated. In summary, we identified three important metabolite-gene pairs, which are LPC 20:4-BCHE, Bisphenol A-SMOC1 and Cortisol-SCIN. There is a very important relationship between metabolites and genes, and we will further explore their connection in subsequent studies.

## 5 Conclusion

In the study, we performed transcriptome and metabolome sequencing analysis on the ovaries of Taihe black-bone silky fowl at the PP and NP, and identified a total of 391 differentially expressed genes and 39 significantly differentially metabolites. Through screening and discussion, we identified five key genes that may affect egg production performance in Taihe black-bone silky fowl: BCHE, CCL5, SMOC1, CYTL1, and SCIN; and three important ovarian significantly differentially metabolites: LPC 20:4, Bisphenol A and Cortisol; through integration analysis of transcriptome and metabolome, we identified three important metabolite-gene pairs: LPC 20:4-BCHE, Bisphenol A-SMOC1 and Cortisol-SCIN. In addition, based on GO and KEGG enrichment analysis, we identified three important pathways that affect egg production performance in Taihe black-bone silky fowls: extracellular region, Neuroactive ligand-receptor interaction and Cytokine-cytokine receptor interaction. This study contributes to a deeper understanding of the regulatory mechanism of egg production in the Taihe black-bone silky fowl and provides a theoretical basis for the improvement of the reproductive performance of the Taihe black-bone silky fowl.

## Data Availability

The data presented in the study are deposited in the https://www.ncbi.nlm.nih.gov/bioproject/PRJNA977820/ repository, accession number PRJNA977820.
