# Supplementary figures and images for: Transcriptomic and metabolomic analyses of the ovaries of Taihe black-bone silky fowls at the peak egg-laying and nesting period
Source: Front Genet. 2023 Oct 9;14:1222087. doi: 10.3389/fgene.2023.1222087 (PMC10591096; doi:10.3389/fgene.2023.1222087)

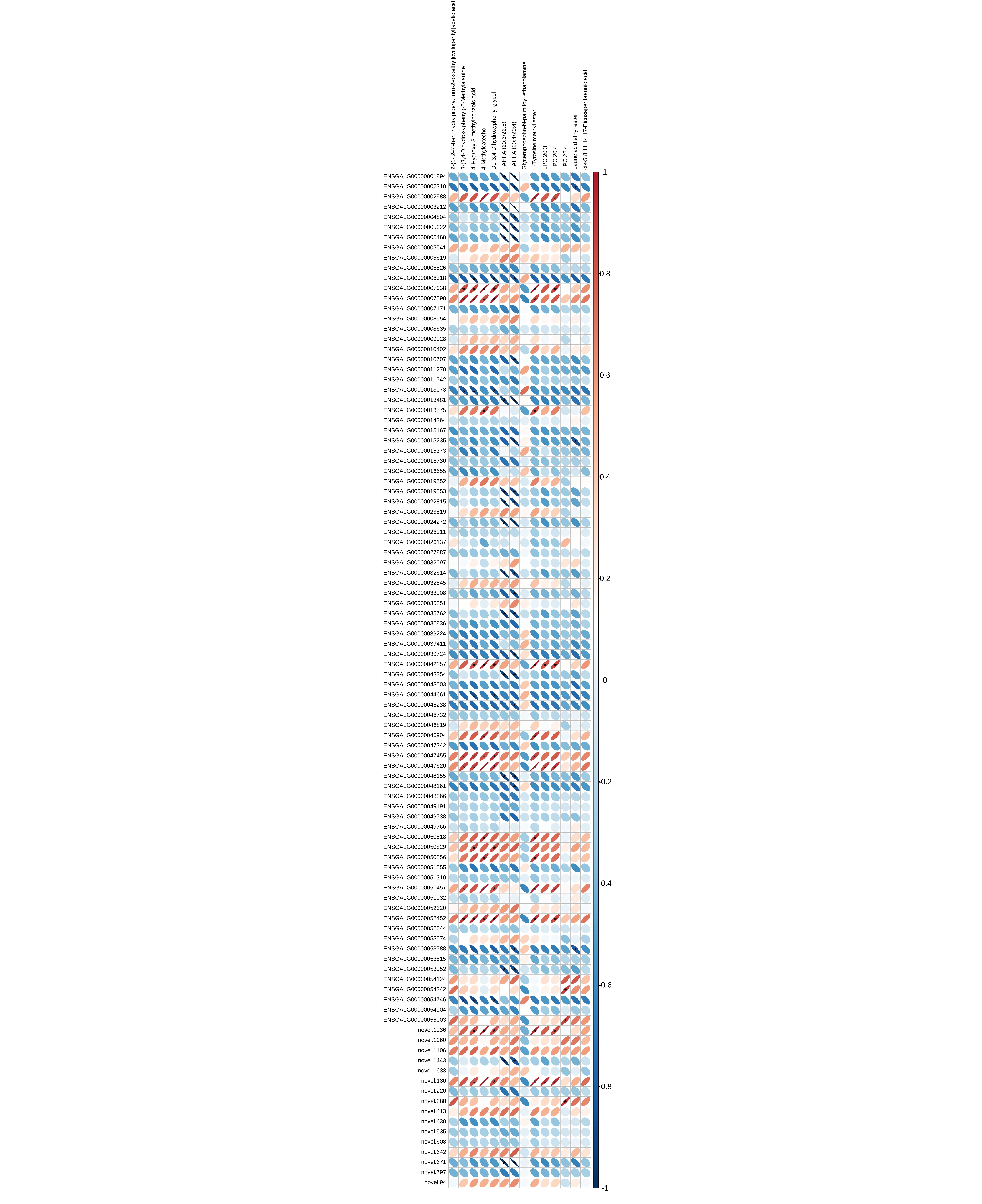

Supplement: Supplementary file 8 [file Image2.PNG]

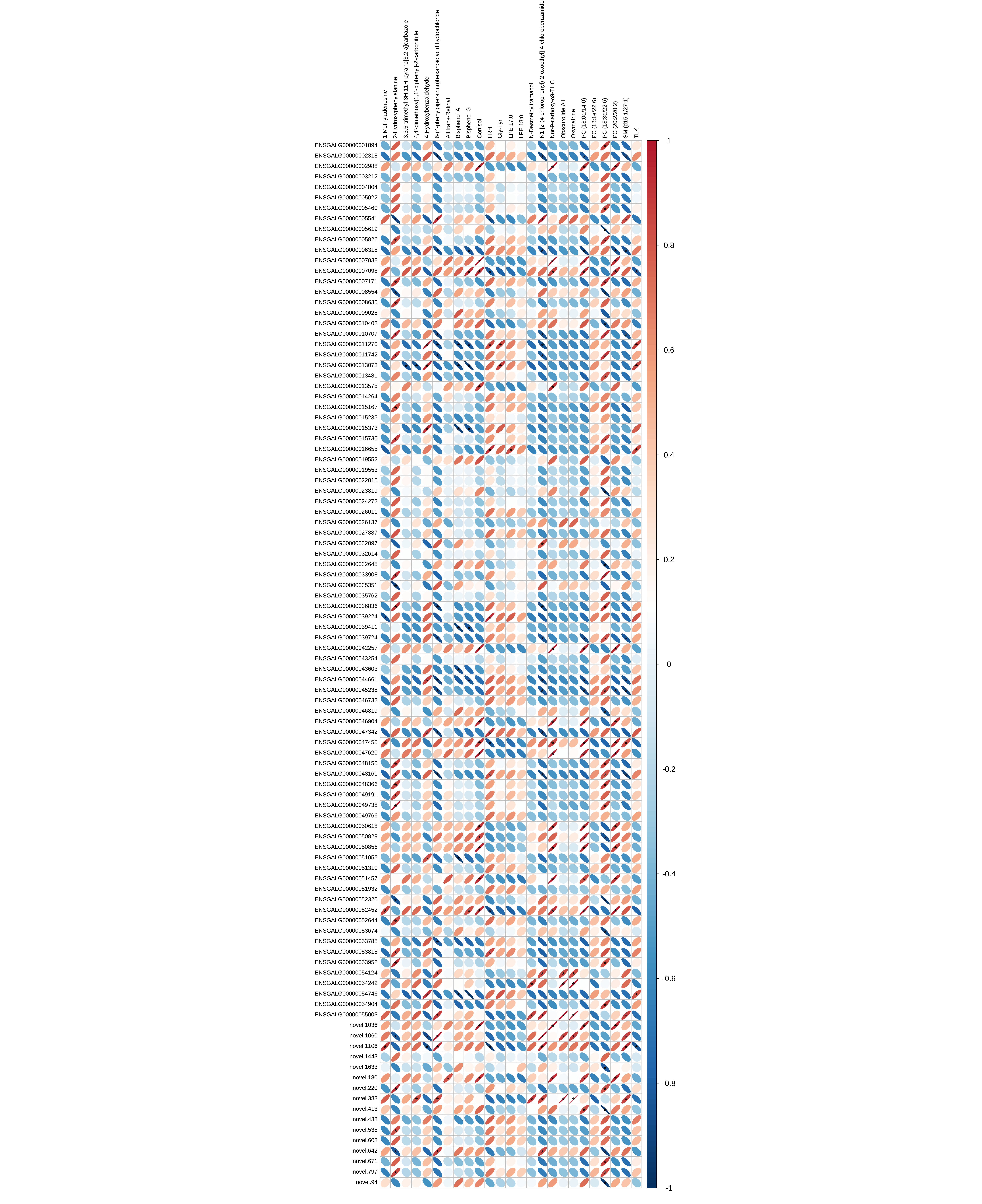

Supplement: Supplementary file 9 [file Image1.PNG]
